# Low birth weight and longitudinal trends of cardiovascular risk factor variables from childhood to adolescence: the bogalusa heart study

**DOI:** 10.1186/1471-2431-4-22

**Published:** 2004-11-03

**Authors:** Maria G Frontini, Sathanur R Srinivasan, Jihua Xu, Gerald S Berenson

**Affiliations:** 1 Department of Public Health, Eastern Virginia Medical School, PO Box 1980, Norfolk VA 23507-1980, USA; 2 Tulane Center for Cardiovascular Health and Department of Epidemiology, Tulane University Health Sciences Center, New Orleans, LA, USA

## Abstract

**Background:**

Several studies have linked low birth weight to adverse levels of cardiovascular risk factors and related diseases. However, information is sparse at a community level in the U.S. general population regarding the effects of low birth weight on the longitudinal trends in cardiovascular risk factor variables measured concurrently from childhood to adolescence.

**Methods:**

Longitudinal analysis was performed retrospectively on data collected from the Bogalusa Heart Study cohort (n = 1141; 57% white, 43% black) followed from childhood to adolescence by repeated surveys between 1973 and 1996. Subjects were categorized into low birth weight (below the race-specific 10^th ^percentile; n = 123) and control (between race-specific 50–75^th ^percentile; n = 296) groups.

**Results:**

Low birth weight group vs control group had lower mean HDL cholesterol (p = 0.05) and higher LDL cholesterol (p = 0.05) during childhood (ages 4–11 years); higher glucose (p = 0.02) during adolescence. Yearly rates of change from childhood to adolescence in systolic blood pressure (p = 0.02), LDL cholesterol (p = 0.05), and glucose (p = 0.07) were faster, and body mass index (p = 0.03) slower among the low birth weight group. In a multivariate analysis, low birth weight was related independently and adversely to longitudinal trends in systolic blood pressure (p = 0.004), triglycerides (p = 0.03), and glucose (p = 0.07), regardless of race or gender. These adverse associations became amplified with age.

**Conclusions:**

Low birth weight is characterized by adverse developmental trends in metabolic and hemodynamic variables during childhood and adolescence; and thus, it may be an early risk factor in this regard.

## Background

The growth of a fetus in an undernourished intrauterine environment is considered to result in adaptive fetal programming or metabolic imprinting with pathophysiologic consequences later in life [1-4]. It is contended that low birth weight at term (<2500 g), a surrogate for impaired gestational environment, is uncommon in industrialized societies, and deprivations that existed before the second world war no longer apply to pregnancies at present [5,6]. In reality, the United States birth data for year 2002 show a prevalence of 7.8% low birth weight, with blacks showing almost twice the rate of whites [7]. Studies world-wide, regardless of socio-economic background, have linked low birth weight to increased risk of insulin resistance, dyslipidemia, hypertension, coronary heart disease, and type 2 diabetes [8-13], although some studies have found a weak or no associations in this regard [5,14-16].

Several studies including our own have examined the association between low birth weight and selected cardiovascular risk factor variables in childhood and adolescence [17-27]. However, information is scant on data linking low birth weight to longitudinal changes of cardiovascular risk factor variables measured simultaneously and serially from childhood to adolescence. As part of the Bogalusa Heart Study, a biracial (black-white) community-based investigation of evolution of cardiovascular risk since childhood [28], the present analysis examines the relationship between low birth weight and the longitudinal trends of adiposity, blood pressure, lipids and lipoproteins, and measures of glucose homeostasis from childhood to adolescence.

## Methods

### Study cohorts

Between 1973 and 1996, 7 cross-sectional surveys of children and adolescents were conducted in the community (65% white, 35% black) of Bogalusa, LA. This panel design, based on repeated cross-sectional examinations performed every 3 to 4 years, resulted in serial observations required for the longitudinal analysis. For the present report, two sets of data were merged as described previously [21]: 1) singleton new born cohort participants (n = 233) whose weights were measured at birth as part of the initial examination during 1973–1974; and 2) singletons (n = 1213) aged 7–11 years who participated in 1987–1988 cross-sectional survey and whose birth weight records were obtained from the Office of Vital Statistics in New Orleans in 1991. Of those with birth weight data (n = 1436), 1329 subjects participated in 2 to 7 surveys of children and adolescents. Exclusion of those with missing data (n = 170), congenital heart disease (n = 11), and diabetes (n = 7) resulted in 1141 eligible subjects (57% white, 47% female).

Low birth weight and control groups were selected from the eligible cohort, according to birth weight percentile cut points [29]. Subjects (n = 123) who had birth weight below the race-specific 10^th ^percentile (whites: <2749 g; blacks: <2438 g) were categorized as low birth weight group; those (n = 296) in the upper normal range of 50–75^th ^percentile (whites: 3402 – 3770 g; blacks: 3133 g – 3487 g) as control group. Birth weights above the 75^th ^percentile were not included in the control group because of the u-shaped associations between birth weight and risk factors or disease [3,30]. Race-specific percentile, rather than World Health Organization (WHO) criterion for low birth weight (<2500 g) was used to define low birth weight because of black-white differences in birth weight distribution [31,32].

### General examination

Identical protocols were used by trained examiners across all surveys [33]. Subjects were instructed to fast for 12 hours prior to screening, and compliance was determined by interview on the morning of examination. Anthropometric and blood pressure measurements were made in replicate and mean values were used.

Height and weight were measured 2 times; subscapular skinfold thickness 3 times. Body mass index (BMI) calculated as weight in kg divided by the square of the height in meters was used as a measure of overall adiposity; subscapular skinfold for truncal fatness. The reproducibility in terms of intraclass (intra-observer) correlation coefficient was greater than 0.99 for weight and height, and greater than 0.97 for subscapular skinfold.

Blood pressure levels were measured in 6 replicates by 2 randomly assigned nurses on the right arm of subjects in a relaxed, sitting position. Systolic and diastolic blood pressures were recorded at the first, fourth, and fifth Korotkoff phases using mercury sphygmomanometer. For this analysis fourth phase was used for diastolic blood pressure because in our experience the fourth phase is more reliably measured in children and more predictive of adult hypertension [34].

### Laboratory analyses

From 1973 to 1986 cholesterol and triglyceride levels in serum were measured using chemical procedures on Technicon Autoanalyzer II (Technician Instrument Corp., Tarrytown, NY). Since then these measurements were done using enzymatic procedures on Abbott VP instrument (Abbott laboratories, North Chicago, IL). Serum lipoprotein cholesterols were analyzed by a combination of heparin-calcium precipitation and agar-agarose gel electrophoresis procedures [35]. Both chemical and enzymatic procedures met the performance requirement of the Lipid Standardization Program of the Centers for Disease Control and Prevention, Atlanta, GA. The laboratory has been monitored for precision and accuracy by the agency's surveillance program since 1973. For example, the average bias in levels of total cholesterol on CDC control samples ranged from -0.1 to -1.6 mg/dL between different cross-sectional surveys, with no consistent pattern over time within or between surveys. The intraclass correlation coefficients between the blind duplicate (10% random sample) values ranged from 0.87 to 0.99 for total cholesterol; 0.88 to 0.99 for triglycerides; 0.86 to 0.98 for LDL cholesterol; and 0.86 to 0.98 for HDL cholesterol.

Plasma immunoreactive insulin levels were measured by a commercial radioimmunoassay kit (Phadebas, Pharmacia Diagnostics, Piscataway, NJ). Plasma glucose was measured by a glucose oxidase method either using a Beckman glucose analyzer (Beckman Instruments, Fullerton, CA) or as part of a multichemistry (SMA20) profile. The intraclass correlation coefficient between blind duplicate values ranged from 0.94 to 0.98 for insulin and 0.86 to 0.98 for glucose. An index of insulin resistance was calculated according to the homeostasis assessment model formula [36]: HOMA-IR=fasting insulin (μu/mL) × fasting glucose (mmol/L) ÷ 22.5.

### Statistical analyses

For test of significance glucose and insulin were logarithmically transformed to approach normality. The average of multiple measurements for subjects within age groups 4 to 11 and 12 to 18 years corresponding to childhood and adolescence periods was used to calculate mean levels of risk variables by birth weight status and age groups. Mean levels of risk variables within each age group were compared between low birth weight and control group by a general linear model, adjusting for age, race, and gender. The longitudinal rates of change in risk variables was assessed by the generalized estimation equation (GEE) method [37] with age as predictor, adjusting for race and gender. Independent association of low birth weight with longitudinal trends of risk variables from childhood to adolescence was assessed by multivariate analysis (GEE). The model included birth weight (low vs control) and risk variables as applicable along with age, age^2^, race and gender and their interaction with birth weight (low vs control). A backward stepwise method was used to remove nonsignificant terms.

## Results

Mean levels of cardiovascular risk variables during childhood (ages 4–11 years) and adolescence (ages 12–18 years) periods are shown in table 1 by birth weight groups. Of the risk variables adjusted for age, race, and gender, levels of HDL cholesterol were significantly lower and LDL cholesterol higher among low birth weight group vs control group during childhood. During adolescence, only glucose levels were significantly higher among low birth weight group vs control group.

**Table 1:** Levels (mean ± SD) of risk variables during childhood and adolescence by birth weight. The Bogalusa Heart Study

Variable	Childhood (4–11 years)		Adolescence (12–18 years)	
	
	Low Birth Weight	Control	Low Birth Weight	Control
BMI (kg/m^2^)	16.7 ± 2.6	17.5 ± 2.9	21.6 ± 5.2	22.7 ± 5.0
Subsc. Skinfold (mm)	8.2 ± 5.1	8.3 ± 6.4	15.9 ± 10.0	16.0 ± 10.7
Syst. BP (mm Hg)	96.4 ± 9.0	98.6 ± 8.1	108.8 ± 9.1	105.2 ± 8.7
Diast. BP (mm Hg)	57.2 ± 10.2	58.7 ± 7.9	66.1 ± 8.0	66.4 ± 7.4
Triglycerides (mg/dL)	62.6 ± 23.2	52.8 ± 20.3	87.1 ± 29.0	83.2 ± 35.3
HDL cholesterol (mg/dL)	44.3 ± 22.6^a^	54.7 ± 17.4	49.9 ± 12.8	51.2 ± 11.5
LDL cholesterol (mg/dL)	76.0 ± 35.4 ^a^	68.6 ± 37.6	99.5 ± 24.7	98.4 ± 24.3
Glucose (mg/dL)	79.7 ± 8.1	80.9 ± 9.7	85.4 ± 8.2^b^	81.6 ± 7.4
Insulin (μu/mL)	8.5 ± 5.7	7.4 ± 4.6	14.8 ± 7.5	13.2 ± 8.6
HOMA-IR	1.7 ± 1.2	1.6 ± 1.0	3.0 ± 2.4	2.7 ± 2.0

Difference between groups (adjusted for age, race, and gender), a: p = 0.05; b: p = 0.02

HOMA-IR: homeostasis model assessment index of insulin resistance

Longitudinal rates of change in cardiovascular risk variables from childhood to adolescence, adjusted for race and gender, are presented in table 2 by birth weight groups. The rate of increase in BMI was significantly lower in low birth weight group compared with control group, while the rate of increase in subscapular skinfold remained similar between the groups. With respect to blood pressure, the rate of increase in systolic blood pressure was significantly higher in low birth weight group than control group. Of the measures of glucose homeostasis, rate of increase of glucose was marginally significant in low birth weight group vs control group. Low birth weight was associated with significantly higher rate of increase in LDL cholesterol; and no significant trends in HDL cholesterol and triglycerides.

**Table 2:** Rates of change in risk variables from childhood to adolescence by birth weight. The Bogalusa Heart Study

Variable	Low Birth Weight	Control	p-value
BMI (kg/m^2^/y)	0.60^†^	0.71	0.03
Subsc. Skinfold (mm/y)	0.91	1.12	0.27
Syst. BP (mmHg/y)	1.70	1.30	0.02
Diast. BP (mm Hg/y)	1.21	1.02	0.26
Triglycerides (mg/dL/y)	2.28	2.92	0.80
HDL cholesterol (mg/dL/y)	-0.53	-0.91	0.24
LDL cholesterol (mg/dL/y)	0.80	0.64	0.05
Glucose (mg/dL/y)	0.50	0.11	0.07
Insulin (μu/mL/y)	0.79	0.67	0.70
HOMA-IR	0.18	0.14	0.44

^†^Regression slope with respect to age in years (y) adjusted for race and gender (generalized equation estimation method).

HOMA-IR: homeostatis model assessment index of insulin resistance.

In a multivariate analysis, low birth weight was retained as an independent predictor variable for adverse longitudinal trends in systolic blood pressure, triglycerides, and glucose (marginal) from childhood to adolescence, regardless of race or gender (table 3). Further, there was a significant interaction between low birth weight and age in this regard, denoting that these variables increased to a greater extent in the low birth weight group than in the control group as individuals became older. An analysis of this data set using the WHO criterion for low birth weight (<2500 g) showed that only 36 subjects were reclassified as having normal birth weight, and the results were essentially the same (data not shown).

**Table 3:** Independent association of low birth weight with longitudinal trends of systolic blood pressure, triglycerides and glucose from childhood to adolescence

Independent Variables Retained	Syst. BP		Triglycerides		Glucose	
	
	β^†^	p-value	β	p-value	β	p-value
Birth Weight (low vs control)	3.84	0.02	48.6	0.08	15.20	0.07
Gender (male vs female)	--	--	--	--	4.31	<0.001
Age	0.39	0.40	12.21	0.01	6.60	<0.001
Age^2^	0.06	0.002	-0.44	0.04	-0.31	<0.001
Insulin	0.11	0.03	1.16	<0.001	0.22	0.02
BMI	0.34	<0.001	--	--	--	--
Birth weight × age	0.45	0.004	12.71	0.03	0.10	0.07
Birth weight × age^2^	--	--	0.55	0.02	2.65	0.10

^†^GEE regression coefficient. The model included birth weight (low vs control) along with age, age^2^, race, and gender, and their interaction with birth weight; and risk variable as applicable.

## Discussion

Information is sparse at a community level in the U.S. general population regarding the effects of low birth weight on the longitudinal trends in C-V risk factor variables measured serially and concurrently from childhood to adolescence. The present community-based study demonstrates the adverse effects of low birth weight on the longitudinal (developmental) trends in systolic blood pressure, triglycerides, and glucose during childhood and adolescence, regardless of race or gender. These observations are in accord with the emerging evidence supporting the concept of intrauterine imprinting and its pathophysiologic consequences enunciated by the fetal origin or thrifty phenotype hypothesis [3].

Many, but not all, previous studies in children and adolescents have found adverse associations between birth weight and levels of cardiovascular risk factor variables [16-27]. In this study, the magnitude of differences in mean levels of cardiovascular risk factor variables between low birth weight and control groups during childhood and adolescence periods were small and nonsignificant for most of the study variables, except for the adverse levels HDL cholesterol and LDL cholesterol in childhood and glucose in adolescence among the low birth weight group. However, in a multivariate analysis of the serial data, the independent adverse effects of low birth weight on the longitudinal trends of systolic blood pressure, triglycerides, and glucose were discernable in the study cohort. Of note, the observed adverse trends associated with low birth weight vs control group were influenced by age in that the differences became greater in magnitude as the children got older. Earlier studies have reported that the inverse associations between birth weight and levels of cardiovascular risk factor variables became stronger with increasing age [26,38]. Whether the potentiating effect of increasing age on low birth weight – risk variable relationship reflects the interaction between fetal programming related to intrauterine malnutrition and the increasing burden with age of unhealthy life-style behaviors including overnutrition and sedentary life style is not clear. In this context, it should be noted that although the rate of yearly increase in BMI, which also includes muscle mass, was significantly lower in low birth weight group, the rate of increase in subscapular skinfold, a measure of truncal fat, remained similar to that of control group. This suggests a gaining of truncal fat, in relative term, over muscle mass in the low birth weight group.

Although observational studies like the present one can not establish causality, several putative mechanisms link low birth weight to adverse trends in risk factor variables. It has been suggested that insulin resistance may be one mechanism by which intrauterine events may program disease risk [39]. Undernutrition in utero is known to cause permanent impairment in growth, structure and function of muscle [39,40], fat [41,42], endocrine pancreas [2,43], liver [30], renal nephrons [44,45] and vasculature [46] due to biologic programming, resulting in insulin resistance/glucose intolerance, hypertension, and dyslipidemia. Further, it has been suggested that intrauterine programming of the hypothalamic-pituitary-adrenal axis may be a functional mechanism underlying the link between low birth weight and above disorders [47], known as components of insulin resistance or metabolic syndrome [48].

This study has certain limitations. The lack of information on the duration of gestation precluded us from adjusting the birth weight for gestational age, a potential confounder. However, earlier studies have found that adverse effects of low birth weight on cardiovascular risk factor variables were independent of gestation period [10,17,49]. Further, it has been pointed out that inclusion of preterm births could actually underestimate these associations [28]. This study also lacks measurements of glucose tolerance and insulin action and secretion used in etiologic studies. Instead, we used the glucose homeostasis measures that are relatively easily measured and applicable at a population level.

## Conclusions

Low birth is characterized by adverse developmental trends in metabolic and hemodynamic variables during childhood and adolescence, especially as the children get older. These observations in conjunction with earlier findings support the view that low birth weight, albeit a crude marker of prenatal growth and physiological environment, is a potential early risk factor for the emergence of metabolic and hemodynamic disorders and related diseases [1,50].

## Abbreviations

BMI, body mass index; LDL, low-density lipoprotein; HDL, high-density lipoprotein

## Competing interests

The author(s) declare that they have no competing interests.

## Authors' contributions

MGF participated in study design, data analysis and manuscript preparation. SRS and GSB contributed to study concept and design, data collection, acquisition of funding and manuscript preparation. JX was involved in measurements of laboratory variables. All authors read and approved the final manuscript.

## Pre-publication history

The pre-publication history for this paper can be accessed here:


